# Design and rationale of the non-interventional, edoxaban treatment in routiNe clinical prActice in patients with venous ThromboEmbolism in Europe (ETNA-VTE-Europe) study

**DOI:** 10.1186/s12959-018-0163-7

**Published:** 2018-05-01

**Authors:** Alexander T. Cohen, Cihan Ay, Philippe Hainaut, Hervé Décousus, Ulrich Hoffmann, Sean Gaine, Michiel Coppens, Pedro Marques da Silva, David Jimenez Castro, Beatrice Amann-Vesti, Bernd Brüggenjürgen, Pierre Levy, Julio Lopez Bastida, Eric Vicaut, Petra Laeis, Eva-Maria Fronk, Wolfgang Zierhut, Thomas Malzer, Peter Bramlage, Giancarlo Agnelli, on behalf of the ETNA-VTE-Europe investigators

**Affiliations:** 1 Guy’s and St Thomas’ NHS Foundation Trust, King’s College London, London, UK; 2 Clinical Division of Haematology and Haemostaseology, Department of Medicine I, Medical University of Vienna, Vienna, Austria; 3 Department of General Internal Medicine, Cliniques Universitaires Saint Luc, UCL, Bruxelles, Belgium; 4 Centre Hospitalier Universitaire de Saint-Etienne, Saint-Priest En Jarez, France; 5 Division of Angiology, Medical Clinic IV, University Hospital, Ludwig-Maximilians-University, Munich, Germany; 6 National Pulmonary Hypertension Unit, Mater Misericordiae University Hospital, Dublin, Ireland; 7 Department of Vascular Medicine, Academic Medical Center, Amsterdam, The Netherlands; 8 Department of Internal Medicine, Arterial Investigation Unit, Hospital de Santa Marta, Lisbon, Portugal; 9 Respiratory Department, Ramón y Cajal Hospital, Madrid, Spain; 10 Division of Angiology, University Hospital Zurich, Zurich, Switzerland; 11 Institute for Health Economics, Steinbeis-University, Berlin, Germany; 12 LEGOS, Université Paris – Dauphine, Paris, France; 13 University of Castilla-La Mancha, Talavera de la Reina, Toledo, Spain; 14 Department of Medicine, Université Paris Descartes, Paris, France; 15 Daiichi Sankyo Europe GmbH, Munich, Germany; 16 Institute for Pharmacology and Preventive Medicine, Berlin, Germany; 17 Internal and Cardiovascular Medicine-Stroke Unit, University of Perugia, Perugia, Italy

**Keywords:** Venous thromboembolism (VTE), Edoxaban, Direct oral anticoagulant (DOAC/NOAC), Anticoagulation, Registry

## Abstract

**Background:**

Venous thromboembolism (VTE, including deep vein thrombosis [DVT] and pulmonary embolism [PE]) has an annual incidence rate of 104–183 per 100,000 person-years. After a VTE episode, the two-year recurrence rate is about 17%. Consequently, effective and safe anticoagulation is paramount. Edoxaban is a direct oral anticoagulant (DOAC) approved VTE treatment. Current safety and efficacy data are derived from clinical trials, and information about treatment durations beyond 12 months are not available.

**Methods:**

ETNA-VTE-Europe is an 18-month prospective, single-arm, non-interventional, multinational post-authorisation safety study. Approximately 310 sites across eight European countries (Austria, Belgium, Germany, Ireland, Italy, the Netherlands, Switzerland and the United Kingdom) will participate in the study, with the intention to represent the regional distributions of centres, healthcare settings and specialties. An estimated cohort of 2700 patients will be recruited, the only enrolment criteria being acute symptomatic VTE, no participation in an interventional study, and treating physician decision to prescribe edoxaban independently from the registry. Data from patient medical records and/or telephone interviews will be collected at baseline, 1, 3, 6, 12 and 18 months. The primary objective is to evaluate the 18-month rate of symptomatic VTE recurrence in patients with VTE treated with edoxaban outside a clinical trial. The co-primary objective is to evaluate the real-world rates of bleeding and adverse drug reactions. Secondary outcomes include rates of other patient-relevant safety events, adherence to and discontinuation of edoxaban. Furthermore, 12-month ETNA-VTE-Europe data will be considered in the context of those for patients receiving different anticoagulants in the PREFER in VTE registry and Hokusai-VTE clinical trial.

**Conclusions:**

ETNA-VTE-Europe will allow the safety and effectiveness of edoxaban to be evaluated over an extended period in acute symptomatic VTE patients encountered in routine clinical practice. Findings will be informative for European practitioners prescribing edoxaban as part of real-world VTE treatment/prevention.

**Trial registration:**

ClinicalTrials.gov Identifier: NCT02943993.

## Background

Venous thromboembolism (VTE), which encompasses both deep-vein thrombosis (DVT) and pulmonary embolism (PE), has an annual incidence rate of approximately 104–183 per 100,000 person-years in individuals of European descent [1]. VTE is the third most common cardiovascular disease. The case-fatality rate is higher after PE than after DVT [2, 3]. Nevertheless, both events increase the probability of subsequent recurrent VTE, which affects approximately 17% of patients at 2 years, indicating that VTE is a chronic disease [4]. Consequently, effective anticoagulation to treat first-time VTE and prevent its recurrence is paramount.

Edoxaban is a direct oral anticoagulant (DOAC), which exerts its effects through inhibition of factor Xa. Based on results from the ENGAGE AF-TIMI 48 and Hokusai-VTE trials [5, 6], it was approved by the European Medicines Agency (EMA) regulatory for two main indications: the prevention of stroke/systemic embolism in patients with non-valvular atrial fibrillation (NVAF) with one or more risk factor, and the treatment/secondary prevention of acute VTE (DVT and/or PE) in adults [7]. However, current evidence for the safety and efficacy of edoxaban is derived from the patient populations enrolled in clinical trials. Furthermore, though current guidelines recommend that anticoagulation following VTE should be continued for at least 3 months and up to an indefinite duration, depending upon the risk of recurrence [8], no data currently exist for treatment with edoxaban beyond 12 months.

As part of an ongoing European risk-management plan, several post-authorisation safety studies (PASS) have been designed to assess the real-world outcomes of edoxaban-treated patients in the EMA-approved indications [9]. Here we describe the design and rationale for the “Edoxaban Treatment in routine cliNical prActice for patients with acute Venous ThromboEmbolism in Europe” (ETNA-VTE-Europe) PASS, the protocol for which has been reviewed and approved by the EMA’s Pharmacovigilance Risk Assessment Committee (PRAC), in accordance with new regulatory requirements. The aim of this PASS is to gain further insight into the safety and effectiveness of treatment and secondary prevention of VTE with edoxaban up to 18 months in routine clinical practice. This study forms part of a global program and safety data will eventually be pooled with ETNA-AF-Europe data and with those from similar Japanese, Thai, Chinese, Taiwanese and Korean regional registries to assess the safety outcomes associated with edoxaban on a worldwide scale.

## Methods

ETNA-VTE-Europe is a single-arm, multinational, prospective, non-interventional PASS, which will be conducted across approximately 310 sites in 8 European countries (Austria, Belgium, Germany, Ireland, Italy, the Netherlands, Switzerland and the United Kingdom). The registry will enrol approximately 2700 patients with acute VTE treated with edoxaban, who will be followed over the 18 months subsequent to the index VTE. Approval from the responsible ethics committees and institutional review boards will be obtained prior to protocol implementation. Informed consent will be obtained from all patients prior to enrolment and compliance with the Declaration of Helsinki will be ensured throughout the study.

### Objectives and outcome measures

The primary objective of ETNA-VTE-Europe is to quantify the long-term rate of VTE recurrence in a large, real-world population of acute symptomatic VTE patients prescribed edoxaban as part of their initial treatment regimen. Accordingly, the primary outcome measure will be the proportion of such patients who experience recurrent symptomatic VTE on one or more occasions within the 18 months following their index VTE, regardless of the length of edoxaban exposure.

The co-primary objective is to collect and evaluate real-world safety data regarding adverse drug reactions (ADRs) in patients treated with edoxaban across the currently approved indications. Therefore, ETNA-VTE-Europe data will be combined with 18-month safety data from the Edoxaban Treatment in routine cliNical PrActice for patients with non-valvular Atrial Fibrillation in Europe (ETNA-AF-Europe) study, a parallel European PASS evaluating the safety and effectiveness of edoxaban in real-world treatment of atrial fibrillation (AF) [10]. The outcome measure will be the proportion of patients who experience death (all-cause, cardiovascular, and VTE-related), bleeding events (major, clinically relevant non-major [CRNM], and minor bleeding; defined according to International Society on Thrombosis and Haemostasis [ISTH] criteria [11]), hepatic events, and/or other ADRs before the 18 month time-point in the two studies.

Secondary objectives are as follows: to evaluate the effects of persistent edoxaban use and permanent discontinuation on the rate of recurrent symptomatic VTE; to assess the rates of patient-relevant safety outcomes, including bleeding, ischaemic/haemorrhagic stroke, systemic embolic events (SEE), post-thrombotic syndrome (PTS), death, and hospitalisation due to cardiovascular causes; and to characterise the extent of adherence to edoxaban, reasons for and rate of discontinuation. To evaluate the effectiveness and safety of edoxaban relative to other anticoagulants, primary and secondary ETNA-VTE outcome data will be appraised in the context of those from the PREFER in VTE real-world registry and Hokusai-VTE clinical trial [12, 13]. The influence of study setting will also be explored by considering real-world ETNA-VTE data in the context of edoxaban data from the Hokusai-VTE clinical trial [12].

### Site selection

In order to represent the regional distributions of centres, healthcare settings, specialties, and approaches to VTE treatment, a sequential site selection process is underway. This involves identification of potential sites, acquisition of relevant institutional details via a feasibility questionnaire, assessment of suitability, and invitation to participate. To be considered eligible, sites are required to have access to acute VTE patients expected to receive edoxaban treatment, the ability to access the Electronic Data Capture (EDC) system and record data in English, and adequate time and staff to perform all study-related documentation activities. They must also agree to complete a screening log with the aim of maximising the potential for consecutive enrolment, and to perform follow-ups according to routine clinical practice. There is an upper limit of 100 patients per site.

### Patient recruitment

The original version of the ETNA-VTE-Europe protocol was approved by the Swiss Agency for Therapeutic Products (Swissmedic) and the central Ethics Committee in Switzerland in February 2015. Accordingly, Swiss sites began enrolling patients at this time. However, protocol revision was necessary due to a change in EMA legislation and a revised PRAC process in July 2015. Upon the approval of the new protocol by the EMA’s PRAC in September 2016, Swiss regulatory bodies were informed of the changes and new approval sought. Patient recruitment according to the revised protocol is now underway, taking place in two waves; the first extends from the last quarter of 2016 to the second quarter of 2018 (Switzerland and Germany) and the second from the first quarter of 2017 to the last quarter of 2018 (all other countries). In each country, the recruitment period will last 2 years, with the aim of obtaining a representative study population of approximately 2700 VTE patients from a variety of healthcare settings.

To be included in the study, patients must have been diagnosed with initial or recurrent acute VTE (distal or proximal DVT and/or PE) that occurred no more than 2 weeks prior to enrolment. In addition, the treating physician must have decided to prescribe the patient edoxaban, with indications that are in line with the edoxaban Summary of Product Characteristics (SmPC). All treatment decisions are independent of the registry and completely at the discretion of the treating physician and/or patient, with no reimbursement for any drugs or therapy received during the study. As such, any concomitant treatment is allowed and changes to medication are unrestricted. The only exclusion criteria are lack of written informed consent and participation in a simultaneous interventional study.

### Documentation and assessment

Data for this study will be sourced from medical records and, when in line with routine clinical practice, from telephone calls between the patient and treating physician. As a non-interventional study, no patient contact, examinations, laboratory tests or procedures are compulsory, and will only be carried out at the discretion of the treating physician, as and when deemed appropriate and without registry influence. A standardised electronic case report form (eCRF) will be completed by the site shortly after enrolment and again after every interaction with a patient over the subsequent 18 months. The eCRFs will be uploaded to the secure, internet-based Medidata Rave EDC system.

Given that the edoxaban SmPC recommends 5 days of parenteral anticoagulation following VTE before starting edoxaban treatment [7], baseline is defined as the first day of heparin/fondaparinux administration after the index acute VTE event. The first data entry, which will be completed following enrolment and within 2 weeks of the index VTE, will include details of baseline patient characteristics, comorbidities, VTE-related parameters, past and current therapy, and other relevant assessments made according to site protocol (Table 1). Memory aids will be provided to all patients at enrolment, with space to record edoxaban-related treatment changes, use of concomitant medication, adverse events, hospitalisation, physician contact, and productivity loss. This aid can be used to assist with recall during subsequent visits/telephone calls, but are voluntary and will not be formally assessed. In each country, all patients will be followed until the last patient for that particular country has completed the 18-month documentation period, in line with EMA requirements. At this point, a last-patient-out (LPO) questionnaire, requesting a minimum of vital status, will be issued for each patient enrolled in the respective country.

**Table 1:** Time points for data assessment

	Baseline*	1 month	3 months	6 months	12 months	18 months	Country-specific LPO
Eligibility criteria^(1)^	X						
Baseline characteristics^(2)^	X						
VTE-related parameters^(3)^	X	X^§^	X^§^	X^§^	X^§^	X^§^	
Edoxaban therapy^(4)^	X	X	X	X	X	X	
Concomitant anticoagulants	X	X	X	X	X	X	
Interventions	X	X	X	X	X	X	
Adherence to VTE therapy^(5)^	X	X	X	X	X	X	
Recurrent VTE^(6)^		X	X	X	X	X	X^†^
Other clinical events^(7)^	X	X	X	X	X	X	
Hospitalisation for cardiovascular disease^(8)^		X	X	X	X	X	
PTS^(9)^	X		X		X	X	
Vital signs^(10)^	X	X	X	X	X	X	
Laboratory parameters^(9)^	X	X	X	X	X	X	
ADRs^(11)^	X	X	X	X	X	X	

*Legend:* VTE, venous thromboembolism; PTS, post-thrombotic syndrome; ADR, adverse drug reaction; LPO, last patient out. ^(1)^ Confirmed first or recurrent VTE within the 2 weeks preceding enrolment; treated with edoxaban according to Summary of Product Characteristics; written informed consent; no simultaneous interventional study participation. ^(2)^ Age and gender, alcohol consumption, smoking status, frailty, comorbidities, and medical history. ^(3)^ Details of past and current VTE risk factors, symptoms, diagnosis, interventions, treatment, and related clinical events (stroke, bleeding events, systemic embolism, non-valvular atrial fibrillation, and malignancies). ^(4)^ History and current status, including dose, prescription intervals, and any changes to edoxaban treatment since last data point (including permanent discontinuation, in which case date, reason and subsequent therapy must be provided). ^(5)^ Physician judgment only. ^(6)^ Timing, diagnosis and interventions. ^(7)^I.e. death, stroke, bleeding events, systemic embolism, non-valvular atrial fibrillation, and malignancies. ^(8)^ Admittance and discharge dates, clinical event, and use of the emergency room and/or intensive care unit. ^(9)^ If assessed. ^(10)^ Including blood pressure, heart rate, height and weight. ^(11)^ As per the Guideline on Good Pharmacovigilance Practices (GVP) Module VI (Management and reporting of adverse reactions to medicinal products; EMA/873138/2011 Rev. 1) [16]; coding according to the standardised Medical Dictionary for Regulatory Activities (MedDRA).*Defined as the first day of heparin administration after the index acute VTE event. ^†^Since 18-month time point. ^§^Changes in symptoms/diagnosis and any other VTE-relevant information

Upon completion of the ETNA-VTE-Europe registry, data will be extracted for the following time points: baseline, 1, 3, 6, 12 and 18 months. Details of the parameters to be evaluated at each time point are outlined in Table 1. The current status, change/events since baseline and change/events since the previous time point will be assessed for each parameter, as appropriate. In addition, recurrence of symptomatic VTE and death since the 18-month time point will be retrospectively documented at the time of LPO per country.

### Definitions

All variables in the ETNA-VTE-Europe study are defined as similarly as possible to those in the PREFER in VTE and Hokusai-VTE studies to permit close comparison of results [6, 13–15]. However, the feasibility of this is somewhat limited by the non-interventional setting. A comparison of the most important variable definitions is provided in Table 2. Permanent edoxaban discontinuation is defined as cessation of edoxaban treatment within the follow-up period.

**Table 2:** Definitions of VTE and bleeding events in ETNA-VTE-Europe, PREFER in VTE and Hokusai-VTE studies

	ETNA-VTE-Europe	PREFER in VTE [13, 14]	Hokusai-VTE [6, 15]
**VTE**			
Baseline	Confirmed first time/recurrent distal/proximal acute symptomatic DVT and/or PE	Confirmed first time/recurrent distal/proximal acute symptomatic DVT and/or PE	Confirmed first time/recurrent **proximal** acute symptomatic DVT and/or PE
Recurrent (during study)	VTE, as adjudicated by an independent CEC	DVT or PE, **as diagnosed by investigator** (**not adjudicated)**	DVT, new non-fatal symptomatic/fatal PE, as adjudicated by an independent CEC^a^
**Bleeding**			
Major	Overt: fatal, symptomatic in a critical area/organ, causing a ≥ 2 g/dL fall in haemoglobin and/or ≥6.0% fall in haematocrit^b^	Overt: fatal, symptomatic in a critical area/organ, causing a ≥ 20 g L^−1^ fall in haemoglobin or **transfusion of ≥ 2 units of whole blood or red cells**^**b**^	Overt: fatal, occurs in a critical site, associated with a ≥ 2 g/dL decrease in haemoglobin or requires a **transfusion of ≥ 2 units of blood**
Life-threatening	Major: intracranial or associated with haemodynamic compromise requiring intervention	**Not specified**	**Not specified**
CRNM	Overt: requires medical attention but does not fulfil major bleeding criteria^b^	Overt: does not meet major bleeding criteria but prompts a clinical response (**hospital admission/physician-guided treatment/change in antithrombotic therapy**)^**b**^	Overt: associated with the need for medical intervention, **contact with a physician, interruption of study drug, or discomfort/impairment of ADL,** but does not fulfil major bleeding criteria
Minor	Overt: other; does not fulfil the criteria for major/CRNM^b^	Overt: other; does not fulfil the criteria for major/CRNM^**b**^	**Not specified**

*Legend:* DVT, deep-vein thrombosis; PE, pulmonary embolism; VTE, venous thromboembolism; CRNM, clinically relevant non-major; CEC, clinical events committee; ADL, activities of daily living. ^a^Members unaware of treatment allocation. ^b^As defined by the International Society of Thrombosis and Haemostasis (2005) [11]. Items in **bold** reflect differences compared to ETNA-VTE-Europe

### Quality control

ETNA-VTE-Europe will be conducted in accordance with the Guidelines for Good Pharmacoepidemiology Practice (GPP) and the EMA Guideline on Good Pharmacovigilance Practices (GVP) Module VIII (management and reporting of adverse reactions to medicinal products; EMA/813938/2011 rev 1) [16, 17]. Automated checks for plausibility and integrity will be carried out at data entry to permit correction or confirmation by the site. Furthermore, approximately 30% of sites will be randomly selected to undergo on-site monitoring, during which random source data verification will be performed. In particular, monitoring activities will closely assess the completeness and correctness of safety data. Patients with missing observations for a particular time point and/or parameter will be omitted from the corresponding analyses.

### Sample size calculations

Assuming an overall 18-month symptomatic VTE recurrence rate [12, 13] of 8.0% in patients who receive edoxaban (regardless of treatment discontinuation) and a relative precision of 15% for a two-sided 95% confidence interval (95% CI), data for 1964 patients will be required to assess with sufficient accuracy the primary outcome. Given an expected dropout rate of 25%, enrolment of 2700 patients should result in an adequate sample size for evaluation at 18 months.

In order to obtain a dataset as large as possible for co-primary outcome assessment, safety data from ETNA-VTE-Europe will be combined with 18-month safety data from ETNA-AF-Europe, a sister European PASS study of edoxaban in approximately 13,100 patients with NVAF [10]. Together, these studies will enrol approximately 15,800 patients, with an overall expected 18-month dropout rate of 20%. Consequently, data for approximately 12,640 patients should be available at 18 months to assess the incidence rates of interest and their 95% CIs. A key aim of this combined analysis is to provide sufficient statistical power to capture uncommon ADRs with low incidence rates; for those that occur at rates of 0.1–1.0%, the corresponding precision of 95% CIs will range from ±0.06% to 0.17%.

### Statistical analysis

Owing to the non-interventional study design, all ETNA-VTE-Europe data analysis will be explorative and descriptive only. In addition to the Europe-wide analysis, safety and effectiveness data will be presented for each individual country and region (Austria, Switzerland and Germany [DACH]; the United Kingdom and Ireland [UK/IE]; Belgium and the Netherlands [BE/NL]; and Italy). Comparisons between patients in particular edoxaban exposure categories (current use, recent use [within the last 3 days], and past use [> 3 days ago], as well as use within the last 30 days) will also be performed, with outcome data additionally presented for all patients permanently discontinuing edoxaban. Furthermore, subgroup analyses comparing patients with/without the following baseline characteristics are planned: renal impairment, hepatic impairment, DVT only (vs. PE with/without DVT), age ≥ 75 years, male gender, initial 30 mg edoxaban dose (vs. 60 mg), and active cancer.

To provide context to our results, the outcomes of ETNA-VTE patients will be appraised relative to those of PREFER in VTE patients treated with a DOAC, PREFER in VTE patients treated with a vitamin-K antagonist (VKA), Hokusai-VTE patients treated with edoxaban, and Hokusai-VTE patients treated with warfarin. These evaluations will be performed on an entirely visual basis, with no joint statistical models planned. Given the one-year follow up of the PREFER in VTE and Hokusai-VTE studies, only 12-month data from ETNA-VTE-Europe will be considered for these purposes [12, 13].

Data will be analysed and presented using descriptive statistics. Categorical variables will be reported as absolute numbers and percentages, and continuous variables will be presented as means with standard deviations or medians with interquartile ranges and minimum/maximum values, alongside the number of missing and non-missing observations. For select variables, 95% CIs will be provided. Where applicable, Kaplan-Meier analysis will be performed to illustrate risk over time. Time-to-event variables will be analysed using Cox proportional hazard regression and presented as hazard ratios with 95% CIs and *p*-values. The type of VTE (DVT only vs. PE ± DVT) will be included in all Cox models as an additional covariate. Based on the results of the Hokusai-VTE and the PREFER in VTE studies [6, 13], no further confounders will be considered at this stage, but any other relevant differences at baseline will be retrospectively added to the model.

A separate analysis combining 18-month safety data from ETNA-VTE-Europe and ETNA-AF-Europe will address the co-primary objective. Data will be presented as absolute and relative frequencies with 95% CIs, overall and for each aforementioned exposure category and subgroup. All statistical analyses will be performed using SAS® version 9.3 or higher (SAS Institute, Cary, NC, USA).

## Discussion

The real-world management of acute VTE has been studied by a number of registries on a national (MASTER and SWIVTER II [18, 19]), European (PREFER in VTE [13]) and global (RIETE, GARFIELD-VTE, RE-COVERY and XALIA [20–23]) scale. Such registries are important because they include patients who are usually omitted from clinical trials due to strict inclusion and exclusion criteria, allowing appraisal of treatment regimes in patients with particular characteristics. Furthermore, large-scale, non-interventional studies permit real-world comparisons across a range of healthcare settings and geographical locations, which may be helpful for evaluating the relative efficiency of national healthcare services and identifying potential improvement strategies. In this sense, ETNA-VTE Europe is similar to existing registries; however, it is different in that it focuses on patients receiving edoxaban, a drug that more recently been approved and is therefore not represented in previous real-world studies. The EMA has reviewed and approved its study protocol. Accordingly, the present registry will add value by providing an as-yet-unexplored insight into the use and associated outcomes of this agent in clinical practice. A comparison between the design of ETNA-VTE Europe and other large-scale VTE-patient registries can be found in Table 3.

**Table 3:** Differences in design between ETNA-VTE Europe and other important European and global observational acute VTE registries

	Study design	Geographical scope	Patients	Agents of interest	Primary objective	Enrolment period	Key outcome measures	Status
ETNA-VTE Europe	Prospective, **single-arm**, observational PASS; **18-M FU**	310 sites across 8 European countries	2700 **edoxaban-treated** acute DVT and/or PE in/outpatients across multiple healthcare settings	**Edoxaban**	To **quantify the real-world, long-term rate of VTE recurrence in patients prescribed edoxaban** as part of acute VTE treatment	**Q4 2016**^**a**^ – **Q4 2018**	Mortality, recurrent VTE, bleeding events, **hepatic events,** ADRs, hospitalisation for CV causes**, edoxaban adherence/discontinuation**	O
RIETE [21]	Prospective, observational study; **3-M FU**	192 sites across 19 countries worldwide (**78% of data from weSpain**)	6855 acute DVT and/or PE in/outpatients across a range of healthcare settings	**Standard therapy (pre-DOACs)**	To improve physician knowledge of the natural history of thromboembolic disease and **develop scores to identify patients at high risk of complications**	**March 2001 – Feb 2004**	Mortality, recurrent VTE, bleeding events	C
XALIA [22, 24]	Prospective, observational PASS; **12-M FU**	Multiple sites^b^in 21 countries **worldwide**	5142 **acute DVT (±PE)** patients at hospitals or community care centres	**Rivaroxaban and standard therapy**	To assess the **safety and effectiveness of rivaroxaban** for the treatment of symptomatic DVT	**June 2012 – March 2014**	Mortality, recurrent VTE, bleeding events, CV events, treatment satisfaction/adherence/discontinuation, healthcare resource utilisation, TEAEs	C
PREFER in VTE [13, 14]	Prospective, observational study; **12-M FU**	381 sites across 7 European countries	3455 acute DVT and/or PE in/outpatients across a range of healthcare settings	**DOACs (pre-edoxaban)**	To explore patient characteristics, VTE management strategies, **healthcare resource usage and associated costs**	**Jan 2013 – Jan 2014**	Mortality, recurrent VTE, bleeding events, MI, stroke, SE, post- thrombotic syndrome, CV events	C
GARFIELD-VTE [20, 25]	Prospective, observational study; **two sequential cohorts; 3-Y FU**	415 sites across 28 countries **worldwide**	10,878 acute DVT and/or PE in/outpatients across a range of healthcare settings	**Standard therapy and DOACs**	To identify **regional/temporal treatment variations** and **assess their impact on** clinical/**economic** outcomes	**July 2014 – Sept 2016**	Mortality, recurrent VTE, bleeding events, post-thrombotic syndrome, chronic thromboembolic pulmonary hypertension, healthcare resource utilisation	O
RE-COVERY [23, 26]	Prospective, observational PASS; **12-M FU**	~17^b^ sites in 5 countries **worldwide**	~ 8000 acute DVT and/or PE patients	**Dabigatran and VKA**	To characterise the VTE patient population and **compare the safety and effectiveness of dabigatran to VKA**	Nov 2015 – Dec 2018	Mortality, recurrent VTE, bleeding events	O

*Legend*: Y, year; M, month; DVT, deep vein thrombosis; FU, follow-up; PE, pulmonary embolism; DOAC, direct oral anticoagulant; VTE, venous thromboembolism; TIA, transient ischaemic attack; PASS, post-authorisation safety study; Q4, fourth quarter; MI, myocardial infarction; SE, systemic embolism; CV, cardiovascular; VKA, vitamin-K antagonist, C, completed; O, ongoing, TEAE, treatment-emergent adverse events. Important differences are highlighted in **bold**. ^a^Swiss sites: February 2015. ^b^Precise number unknown

Current evidence for the safety and efficacy of edoxaban largely emanates from the phase III Hokusai-VTE clinical trial [6]. A key limitation of this study was the exclusion of patients with characteristics encountered in routine clinical practice, such as those with isolated distal symptomatic DVT, a creatinine clearance of < 30 mL/min, and significant liver disease. Consequently, the effectiveness and safety in some clinical subgroups to edoxaban remain unrepresented and uncharacterised. Data gathered from the ETNA-VTE-Europe study with minimal selection criteria will address this knowledge gap, with several pertinent subgroup analyses already planned. The gathered data will also provide a snapshot of the types of patients being prescribed edoxaban, the doses used, and the durations of treatment in a real-life setting. This will permit appraisal of the appropriateness of such therapeutic decisions as well as acting as a marker for evaluation of subsequent trends of prescription.

An additional limitation of current studies is that data are only available for up to 12 months of edoxaban therapy [6]. Considering that a number of patients will require longer-term anticoagulation in clinical practice [8], those continuing edoxaban dosing beyond 12 months will find themselves in uncharted waters. The 18-month follow-up period planned for ETNA-VTE-Europe patients will provide clinicians and patients with valuable information regarding the safety and net clinical benefit of extended treatment. In particular, trends in rare ADRs such as liver events and major bleeding will be of interest, given their low frequency in the Hokusai-VTE trial [6]. Combination of ETNA-VTE-Europe data with those from the ETNA-AF-Europe sister study will provide an even larger population from which to assess these safety outcomes [10]. Thus, the present PASS is a responsible and important part of the early post-marketing phase, designed to address several of the knowledge gaps highlighted in the EMA risk-management plan for edoxaban [9].

The single-arm design of the ETNA-VTE-Europe study reflects its key aim of characterising the real-world safety and effectiveness of edoxaban. However, visually comparing our results to those from similar studies with different anticoagulants will help to provide perspective. To these ends, data from the recent PREFER in VTE real-world registry will be used to contextualise data from ETNA-VTE-Europe, selected for its comparable bleeding definitions, coinciding data assessment time points, and high patient numbers (3545 patients) [14]. This will allow us to gain an idea of the real-world safety and effectiveness of edoxaban relative to agents such as VKAs and other DOACs, not only in the overall population, but also in specific patient subsets, such as those with renal or hepatic impairment. Furthermore, several of the countries and regional delineations in ETNA-VTE-Europe (such as Italy, DACH and UK/IE) also feature in the PREFER in VTE study, enabling various approximate geographical comparisons [13]. A further evaluation of ETNA-VTE-Europe real-world results in the context of those from the Hokusai-VTE clinical trial will provide insight into the influence of stringent enrolment criteria and closer monitoring on edoxaban-related outcomes. This will indicate to what extent the encouraging findings from phase III studies can be expected to translate into clinical practice.

### Potential limitations

As a prospective, non-interventional registry that aims to represent everyday occurrence and management of VTE, the stringent inclusion/exclusion criteria, physician/patient blinding, and site/protocol standardisation that typify RTCs do not apply. This introduces the possibility for biases that are difficult to avoid, measure or correct for. In particular, heterogeneous timing and length of edoxaban exposure may prove problematic for the interpretation of results, thought this will be taken into consideration at data analysis. Furthermore, in such a large-scale study, some incompleteness, insufficient detail, or inaccuracy of patient data is unavoidable; however, measures such as the careful design of case report forms and data monitoring/verification aim to minimise such occurrences. Despite the inherent limitations associated with observational registries, the all-inclusive nature of ETNA-VTE Europe may be seen as an advantage for obtaining a realistic snapshot of the clinical climate, with two-wave recruitment potentially allowing changes in treatment behaviour over time to be elucidated. Finally, while the single-arm nature of the study means that direct comparisons with other anticoagulants will not be possible, close accordance between the designs of PREFER in VTE and ETNA-VTE Europe should permit a reasonable appraisal of outcome differences.

## Conclusions

The international, observational ETNA-VTE-Europe registry will allow the safety and effectiveness of edoxaban in the general population of acute symptomatic VTE patients to be assessed over an extended time period. The planned series of explorative analyses will provide essential information regarding edoxaban treatment in a range of as-yet-unrepresented patient subsets, the effect of treatment duration and discontinuation on outcomes, and the relative costs/benefits associated with edoxaban use compared to that of other commercially available anticoagulants. Findings will help to elucidate whether the good safety profile observed in the Hokusai-VTE clinical trial can be extrapolated to the real-world population, as well as providing a wealth of valuable information for European practitioners prescribing or contemplating the use of edoxaban as part of VTE treatment/secondary prevention.

## Appendix

### List of ETNA in VTE investigators

#### Austria

Cihan Ay, Allgemeines Krankenhaus Medizinische Universität Wien, Wien; Matthias Hoke, AKH - Medizinische Universität Wien, Wien; Marianne Brodmann, LKH - Universitätsklinikum Graz, Graz; Rudolf Kirchmair, Medizinische Universität Innsbruck, Innsbruck; Rainer Mathies, Landeskrankenhaus Feldkirch, Feldkirch; Markus Rauter, Klinikum Klagenfurt am Wörthersee, Klagenfurt; Klemens Reinberg, Gemeinschaftspraxis Wachau, Weißenkirchen in der Wachau; Vinzenz Stepan, Krankenhaus der Elisabethinen Graz, Graz; Thomas Maca, Ordination Dr. Maca, Wien; Hans Domanovits, AKH - Medizinische Universität Wien, Vienna; Wolfgang Salmhofer, Landeskrakenhaus - Universitaetsklinikum Graz, Graz; Thomas Weiss, Wilhelminenspital der Stadt Wien, Wien; Walter Klimscha, Sozialmedizinisches Zentrum Ost - Donauspital, Vienna; Alexander Kober, Ordination, Sankt Aegyd am Neuwalde; Franz Hinterreiter, Konventhospital Barmherzige Brüder, Linz; Johann Auer, KH St. Josef Braunau, Braunau; Armaghan Gomari-Grisar, Krankenhaus Barmherzige Schwestern Wien, Wien; Joeg Jabkowski, Krankenhaus der Elisabethinen Linz, Linz; Paul Pinter, Wahlarzt Ordination, Dr. Paul Pinter, Altenmarkt an der Triesting

#### Belgium

Philippe Hainaut, Cliniques Universitaires Saint-Luc, Bruxelles; Laure Gilis, CHC - Clinique St-Jospeh, Liège; Herman Schroe, Ziekenhuis Oost-Limburg, Genk; Jos Vandekerkhof, Jessa Ziekenhuis - Campus Virga Jesse, Hasselt; Pascal Vranckx, Jessa Ziekenhuis Hospital, Hasselt; Peter Verhamme, UZ Leuven, Leuven; Mikhael Janssen, AZ Groeninge - Kennedylaan, Kortrijk; Guy Vereecken, Vereecken Guy, Halen; Luc De Munck, De Munck, Vilvoorde; Bernard Vandooren, Algemeen Ziekenhuis West, Veurne; Johan Duchateau, AZ Sint-Maarten, Duffel; Jan De Letter, AZ Sint-Jan Brugge, Brugge; Muriel Lins, AZ Sint-Maarten, Duffel; Jean-Claude Wautrecht, ULB Hopital Erasme, Bruxelles; Marc Delforge, CHR de Huy, Huy; Philippe Hermans, CHU Saint-Pierre, Bruxelles; Philippe Borgoens, CHR Citadelle, Liege; Jean-Baptiste Nicolas, CHU UCL Namur, Yvoir; Geoffrey Debonnaire, Sint-Elisabeth Ziekenhuis, Zottegem; Marion Delcroix, UZ Leuven, Leuven; Stéphane Vanden Bemden, bvba Medisch Kabinet Dr. Vanden Bemden, Steenokkerzeel; Jaak Mortelmans, Mortelmans, Jaak, Oostham; Muriel Sprynger, CHU de Liege, Liège; Yohan Balthazar, SPRL MG Balthazar & Ballard, Natoye; Geert Vileyn, Huisartsenpraktijk Vileyn bvba, Blankenberge; Michel Goetinck, Huisartsenpraktijk De Stoute, Brugge; Bart Segers, Wichelen; Philippe De Vleeschauwer, Heilige Hart Ziekenhuis Lier, Lier; Michel De Pauw, Universitair Ziekenhuis Gent, Gent; Karen Wustenberghs, GZA Ziekenhuizen - Campus Sint-Augustinus, Wilrijk; Roeland Dierickx, Huisartsen Dierickx, Westouter

#### Germany

Dirk Härtel, Klinikum Lippe GmbH, Detmold; Gunter Hergdt, Praxis Dr. med. Gunter Hergdt, Obermichelbach; Holger Michel, Gemeinschaftspraxis Michel und Leffler, Lutherstadt Eisleben; Annegret Otto, Gemeinschaftspraxis Dres. med. Stenzel, Ebert & Otto, Riesa; Andreas Rieker, Facharztpraxis Rieker Dr. med. Andreas Rieker - Venenpraxis im “Gesundheitszentrum Südstraße”, Lauffen am Neckar; Toralf Schwarz, INFO-MED Leipzig GmbH, Markkleeberg; Jens Taggeselle, Kardiologische Praxis Dr. Jens Taggeselle, Markkleeberg; Oliver Gastmann, Ilm-Kreis-Kliniken Arnstadt-Ilmenau gGmbH, Arnstadt; Diana Razavi, Praxis Diana Razavi, Steinau; Petra Herrmann, Praxis Dipl.- Med. Petra Herrmann, Eisenach; Dag-Alexander Keilhau, Medizinisches Versorgungszentrum für Innere Medizin, Hamburg; Bernd-Thomas Kellner, Gemeinschaftspraxis Dres. Niels Erhardt und Bernd-Thomas Kellner, Dornburg-Camburg; Diethard Predel, Praxis für Gefäßmedizin, Nordhausen; Rainer Rippert, Praxis Dr. Rippert, Wiesbaden; Reinhardt Sternitzky, Zentrum für klinische Prüfungen in der Facharztzentrum Dresden-Neustadt GbR, Dresden; Andor Schmidt, Omdas GmbH and Praxis Dr. med. Andor Schmidt, Offenbach; Matthias Schulze, Asklepios Klinikum Schwalmstadt, Schwalmstadt; Heiko Schneider, Medigra - Gemeinschaftspraxis Dres. Heiko Schneider & Peter Rostock, Apolda; Franz Wolf, Praxis Dres. Wolf, Straubing; Andreas Greve, Innere Medizin im Gesundheitszentrum, Ahrensburg; Matthias Ulrich, Angiologie am Coppiplatz, Leipzig; Norbert Schön, Kardiologie Mühldorf am Inn, Mühldorf; Günter Rehling, Gemeinschaftspraxis Dres. Michael Eis und Günter Rehling, Sand am Main; Bernhard Witzenbichler, HELIOS Amper-Klinikum Dachau, Dachau; Bernadett Brado, Praxis für Angiologie, Hämatologie, Innere Medizin Dr. med. Bernadett Brado, Heidelberg-Neuenheim; Thomas N. Abahji, Ärztehaus GerMedicum, Germering; Johannes Brachmann, Klinikum Coburg GmbH, Coburg; Hans-Walter Bindig, Praxis Dr. Bindig, Georgensgmünd; Siamak Pourhassan, Gemeinschaftspraxis für Gefäßchirurgie/ Gefäßmedizin - Drs. Heim & Pourhassan, Oberhausen; Tobias Geisler, Deutsches Herzkompetenzzentrum am Universitätsklinikum Tübingen, Tübingen; Berthold Amann, Franziskus-Krankenhaus Berlin, Akademisches Lehrkrankenhaus der Charité, Berlin; Svetlana Tlechas-Tkatsch, Praxis S. Tlechas-Tkatsch, Potsdam; Markus Stücker, St. Maria-Hilf-Krankenhaus, Bochum-Gerthe; Rainer Schmiedel, Praxis Dr. med. Rainer Schmiedel, Kaiserslautern; Ernst G. Vester, Evangelisches Krankenhaus Düsseldorf, Düsseldorf; Janna Dshabrailov, Praxis Dr. Dshabrailov, Osnabrück; Ayham Al-Zoebi, Kardiologische Praxis Dr. Al-Zoebi, Wermsdorf; Sylvia Baumbach, Gemeinschaftspraxis Dr. med. Sylvia Baumbach und Dr. Elke Reichelt, Apolda; Wilma Grosskopf, Gemeinschaftspraxis Dres. Josef Anton Großkopf und Wilma Großkopf, Wallerfing; Michael Czihal, Klinikum der Universität München, München; Christoph Hehrlein, Universitäts-Herzzentrum Freiburg Bad Krozingen GmbH, Freiburg; Thomas Ludwig, Praxis Dr. med. Thomas Ludwig, Farchant; Daniel Kretzschmar, Universitätsklinikum Jena, Jena; Teja Müller, Praxis Innere Medizin Dr. Teja Müller, Wehrheim; Harriet Simone Werno, Parcside medical center, Nuernberg; Julio Perez-Delgado, Arberlandklinik Viechtach, Viechtach; Edelgard Lindhoff-Last, CCB - Cardioangiologisches Centrum Bethanien, Frankfurt; Thomas Schmitz-Rixen, Universitätsklinikum Frankfurt, Frankfurt am Main; Oliver Ritter, Städtisches Klinikum Brandenburg, Brandenburg an der Havel; Thomas May, Krankenhaus Porz am Rhein gGmbH, Köln; Enno Eißfeller, Praxis Dr. med. Eißfeller, Woellstein; Klaus Fenchel, MVZ MP Saaletal, Saalfeld; Marcus Thieme, MEDINOS Kliniken des Landkreises Sonneberg GmbH, Sonneberg; Christian Heiß, Universitätsklinikum Düsseldorf, Düsseldorf; Thomas Bader, Praxis Dr. med. Bader, Heilbronn-Biberach; Rainer J. Zotz, Marienhausklinikum Eifel Bitburg, Bitburg; Christoph Kalka, Marienhospital Brühl GmbH, Brühl; Johannes Haas, CIMS UG mbh, Bamberg; Andreas Hagenow, Praxis Dr. med. Andreas Hagenow, Elsterwerda; Karl-Heinz Binias, AMEOS Klinikum Schönebeck, Schönebeck; Jan Beyer-Westendorf, Universitätsklinikum Carl Gustav Carus TU Dresden, Dresden; Veselin Mitrovic, Kerckhoff-Klinik Forschungsgesellschaft mbH, Bad Nauheim; Wolfram Oettler, Praxis für Gefäßmedizin, Görlitz; Claudia Zemmrich, MVZ Dres. Ramdohr - Praxis für Cardiovascular und Ultraschalldiagnostik, Berlin; Jaswant Singh, St. Elisabeth-Krankenhaus, Jülich; Matthias Leschke, Klinikum Esslingen GmbH, Esslingen a. N.; Thomas Herrmann, Fachinternistische Gemeinschaftspraxis Weinheim, Weinheim; Stefan Hetzel, Gemeinschaftspraxis Dres. Hetzel & Grewe-Kemper, Greven; Frank Hamann, Klinikum Konstanz, Konstanz; Christina Hart, Universitätsklinikum Regensburg, Regensburg; Matthias Tenholt, Theresienkrankenhaus und St. Hedwig-Klinik GmbH, Mannheim; Martin Andrassy, Fürst-Stirum-Klinik Bruchsal, Bruchsal; Andreas Wilke, Kardiologische Praxis Papenburg Dr. Hans-Jürgen Stühn-Pfeifer und Dr. Andreas Wilke, Papenburg; Friederike Baron-Gielnik, CardioMed an der Alster, Hamburg; Muwafeg Abdel-Qader, Praxis Dr. med. Abdel-Qader, Winsen; Rainer Hennecke, Dres. Rolf Schenk und Rainer Hennecke, Winsen; Birgit Gerecke, MVZ Ambulantes Kardiologisches Zentrum Peine, Peine; Uwe Gremmler, MVZ Ambulantes Kardiologisches Zentrum Peine, Peine; Volker Eissing, MVZ Birkenallee GmbH, Papenburg; Nikolaos Proskynitopoulos, Kardiologische Gemeinschaftspraxis, Nienburg; Stefan Regner, Gemeinschaftspraxis Mainz Mitte am Gesundheitscentrum Mainz, Mainz; Heiner Müller Heiner, Berufsausübungsgemeinschaft Dres. med. Kolitsch/Müller, Katzhütte; Stephan Lüders, St. Josefs-Hospital, Cloppenburg; Elvira Maria Lembens, Praxis Dres. Christoph Lembens und Elvira Maria Lembens, Mainz; Werner Jung Werner, Schwarzwald-Baar Klinikum Villingen-Schwenningen GmbH, Villingen-Schwenningen; Frank Heckmann, Gefäßzentrum Dr. Heckmann und Kollegen, Neckargemuend; Kurt Jocham, Dialysezentrum Memmingen - Internistisches Facharztzentrum, Memmingen; Ralf Berg, Praxis im alten Rathaus, Einzelpraxis Dr. med. R. Berg, Ühlingen-Birkendorf; Thomas Dengler, Kreiskrankenaus am Plattenwald, Bad Friedrichshall; Uwe Zwettler, Atos clinic Heidelberg, Dres. Heckmann/Rieger, Heidelberg; Martin Rieger, Zentrum für Gefäßerkrankungen und Präventivmedizin, Neckargemünd; Pascal Bauer, UKGM - Universitätsklinikum Gießen und Marburg GmbH, Gießen; Mirko Böhme, Praxis Dr. Mirko Böhme, Sulzberg; Christian Loges, Drs. Haney/Gehrig/Loges, Mosbach; Sebastian Kruse, Gemeinschaftspraxis Kruse / Kuth, Aachen-Walheim; Clemens Tebbe, Marien-Hospital Euskirchen, Euskirchen; Oliver Bruder, Elisabeth Krankenhaus Essen, Essen; Dietrich Gulba, St. Marien Hospital, Oberhausen; Norbert Ludwig, Gemeinschaftspraxis Prof.Dr. Ludwig, Dr. Honl, Willich; Christoph Nielen, Gemeinschaftspraxis für Gefäßmedizin GbR, Mönchengladbach; Ulrich Overhoff, Zentrum fuer Praevention und Rehabilitation, Siegen; Helmut Renz, Gemeinschaftspraxis Vogelstang, Mannheim; Holger Lawall, Gemeinschaftspraxis Prof. Dr. med. Curt Diehm und Dr. med. Holger Lawall, Ettlingen; Sabine Genth-Zotz, Katholisches Klinikum Mainz, Caritas Werk St. Martin Gem. Träger und Betriebsf. GmbH, Mainz; Peter Wirtz, Kreiskrankenhaus Mechernich GmbH, Mechernich; Dirk Henning Walter, Kardiologie am Tibarg, Hamburg; Astrid Schmidt-Reinwald, Praxis Dr. med. Astrid Schmidt-Reinwald, Trier

#### Ireland

Brian McCullagh, Mater Private Hospital, Dublin; Mike Watts, University Hospital Limerick, Limerick; Azhar Bhatti, Connolly Hospital, Dublin; Peter Branagan, Beaumont Hospital,Dublin; Stuart Carr, Blackrock Clinic, Dublin

#### Italy

Michele Dalla Vestra, Ospedale dell’Angelo, Mestre; Crisitiano Bortoluzzi, Ospedale civile SS. Giovanni e Paolo, Venezia; David Imberti, Presidio Ospedaliero di Piacenza Ospedale “Guglielmo Da Saliceto”, Piacenza; Domenico Prisco, Azienda Ospedaliera Universitaria Careggi, Firenze; Serena Rupoli, Azienda Ospedaliero Universitaria Ospedali Riuniti di Ancona "Umberto I, G.M. Lancisi, G. Salesi", Ancona; Maria Amitrano, Azienda Ospedaliera San Giuseppe Moscati, Avellino; Crisitiana D’Amrosio, Ospedale Ferdinando Veneziale, Isernia; Angelo Ghirarduzzi, Azienda Ospedaliera Arcispedale Santa Maria Nuova - IRCCS, Reggio Emilia; Giovanni Di Minno, Azienda Ospedaliera Universitaria Federico II, Napoli; Andrea Fontanella, Ospedale Buonconsiglio - Fondazione Fatebenefratelli, Napoli; Maria Teresa De Denato, Azienda Ospedaliera Universitaria S. Giovanni di Dio - Ruggi D’Aragona, Salerno; Sophie Testa, ASST di Cremona - Ospedale di Cremona, Cremona; Lorenzo Malatino, Azienda Ospedaliera per l’emergenza “Canizzaro”, Catania; Francesca Corsini, Ospedale Sant’Andrea, La Spezia; Rodolfo Tassara, Presidio Ospedaliero di Savona - Cairo Montenotte Ospedale San Paolo, Savona; Gabriele Giordano, Villa dei Fiori - Casa di cura privata, Acerra; Anita Carlizza, Azienda Ospedaliera San Giovanni Addolorata, Roma; Riccardo Margheriti, Presidio Ospedaliero Giovan Battista Grassi, Roma; Fausto Marrocco, Presidio Ospedaliero Santa Scolastica, Cassino; Raffaele Landolfi, Fondazione Policlinico Universitaria “A.Gemelli”, Roma; Sabina Villalta, Ospedale di Treviso, Treviso; Adriana Visona, Presidio Ospedaliero San Giacomo Apostolo, Castelfranco Veneto; Egidio Imbalzano, Azienda Ospedaliera Universitaria Policlinico “G. Martino”, Messina; Maurilio Cirrito, Fondazione Istituto “G. Giglio”, Cefalù; Massimo Arquati, Presidio Ospedaliero Sacco, Milano; Giampiero Avruscio, Azienda Ospedaliera di Padova - Policlinico, Padova; Ginacarlo Agnelli, Azienda Ospedaliera di Perugia, Perugia; Lucia Anna Mameli, Ospedale Civile SS Annunziata A.S.L.1 Sassari, Sassari; Armando D’Angelo, Ospedale San Raffaele IRCCS, Milano; Roberta Risso, Ospedale Santo Spirito di Bra, Cuneo; Alessandro Celi, Azienda Ospedaliera Universitaria Pisana - Ospedale di Cisanello, Pisa; Vieri Vannucchi, Presidio Ospedaliero Santa Maria Nuova, Firenze; Fernando Parente, Ospedale Vito Fazzi, Lecce; Francesco Ventrella, Presidio Ospedaliero G. Tatarella, Cerignola; Francesco Dentali, Ospedale di Circolo e Fondazione Macchi, Varese; Pietro Tropeano, Ospedale Santa Maria degli Angeli, Pordenone; Pasquale Morella, Azienda Ospedaliera A. CARDARELLI, Napoli; Aldo Salvi, Ospedali Riuniti Ancona, Ancona; Gabriele Frausini, Azienda Ospedaliera MARCHE NORD Presidio Ospedaliero Santa Croce, Fano; Roberto Catalini, Ospedale Generale Provinciale, Macerata; Mauro Campanini, Azienda Ospedaliera Universitaria Maggiore della Carità, Novara; Corrado Amato, Policlinico Universitario Palermo, Palermo; Monica Demarco, Humanitas Mater Domini, Castellanza; Iolanda Enea, Ospedale S. Anna e S. Sebastiano, Caserta; Nello Zanatta, Ospedale S. Maria dei Battuti, Conegliano Veneto; Rita Pepe, Ospedale S. Eugenio, Roma; Roberto Cappelli, Azienda Ospedaliera Universitaria Senese, Siena; Stefano Barolo, Ospedale S. Lazzaro, Alba; Giuseppe Guigliano, Azienda Ospedaliera Universitaria Federico II, Napoli; Beilde Cosmi, Ospedale Sant’Orsola - Malpighi, Bologna; Domenic Blasis, Ospedale SS Filippo e Nicola, Avezzano; Jeness Simona Campodonico, Centro Cardiologico Monzino, Milano; Franco Spangaro, Ospedale Cattinara, Trieste

#### Netherlands

Michiel Coppens, Academisch Medisch Centrum, Amsterdam; Albert Mairuhu, HagaZiekenhuis, Den Haag; Wim Boersma, Noordwest Ziekenhuisgroep, Alkmaar; Marije ten Wolde, Flevoziekenhuis, Almere; Houshang Monajemi, Rijnstate, Arnhem; Heike Noordzij-Nooteboom, Van Weel-Bethesda Ziekenhuis, Dirksland; Gert-Jan Braunstahl, Franciscus Gasthuis, Rotterdam; Ragnar Lunde, Laurentius Ziekenhuis, Roermond; Sanne van Wissen, Onze Lieve Vrouwe Gasthuis, Locatie Oost, Amsterdam; Adriaan Dees, Ikazia Ziekenhuis, Rotterdam

#### Switzerland

Marc Righini, Hopital Universitaire Geneve, Geneva; Lucia Mazzolai, Centre Hospitalier Universitaire Vaudois, Lausanne; Frederic Baumann, Universitaetsspital Zuerich, Zuerich; Marc Schindewolf, University of Bern, Bern; Daniel Staub, Universitaetsspital Basel, Basel; Juerg H. Beer, Kantonsspital Baden AG, Baden; Martin Banyai, Cantonal hospital Lucerne, Luzern; Stefan Kradolfer, Facharzt FMH ALLG. Medizin, Basel; Daniel Erni, Schwanenpraxis, Luzern

#### United Kingdom

Karen Breen, Guy’s Hospital, London; Ewart Jackson-Voyzey, Axbridge and Wedmore Medical Practice, Axbridge; Piers Clifford, Wycombe Hospital, High Wycombe; Peter MacCallum, Barts Health NHS Trust, London; Azhar Zafar, Danes Camp Medical Practice, Northampton; Anja Drebes, Royal Free London NHS Foundation Trust, London; Asok Venkataraman, George Eliot Hospital, Nuneaton; Gunaratnam Gunathilagan, Queen Elizabeth the Queen Mother Hospital, Margate; Salah Matti, Basingstoke and North Hampshire Hospital, Basingstoke; Nicola Stevenson, Arrowe Park Hospital, Wirral; Tamara Everington, Salisbury District Hospital, Salisbury; James Uprichard, St George’s Hospital, London; Anand Dixit, Royal Victoria Infirmary, Newcastle upon Tyne; Piers Clifford, Hammersmith Hospital, London; Tina Dutt, Royal Liverpool University Hospital, Liverpool; Jeremy Platt, The Binfield Surgery, Binfield; Mark Richardson, Lindum Medical Practice, Lincoln; Paul Gilbert Conn, Ballygomartin Group Practice, Belfast; Charalampos Kartsios, Birmingham Heartlands Hospital, Birmingham; Muhsin Almusawy, Bedford Hospital, Bedford; Margaret Bowers, Ulster Hospital South Eastern Health and Social Care Trust, Belfast.

## Abbreviations

ADR: Adverse drug reaction
BE/NL: BELGIUM/the Netherlands
CI: Confidence interval
CRNM: Clinically relevant non-major bleeding
DACH: Austria, Switzerland and Germany
DOAC: Direct oral anticoagulant
DVT: Deep vein thrombosis
eCRF: Electronic case report form
EDC: Electronic data capture
EMA: European Medicines Agency
GPP: Good Pharmacoepidemiology Practice
GVP: Good Pharmacovigilance Practices
LPO: Last patient out
NVAF: Non-valvular atrial fibrillation
PASS: Post-authorisation safety study
PE: Pulmonary embolism
PTS: Post-thrombotic syndrome
SEE: Systemic embolic events
UK/IE: United Kingdom/Ireland
VTE: Venous thromboembolism

## References

[1] Heit JA (2015). Epidemiology of venous thromboembolism. Nat Rev Cardiol.

[2] Goldhaber SZ, Bounameaux H (2012). Pulmonary embolism and deep vein thrombosis. Lancet.

[3] Cushman M, Tsai AW, White RH, Heckbert SR, Rosamond WD, Enright P, Folsom AR (2004). Deep vein thrombosis and pulmonary embolism in two cohorts: the longitudinal investigation of thromboembolism etiology. Am J Med.

[4] Heit JA (2012). Predicting the risk of venous thromboembolism recurrence. Am J Hematol.

[5] Giugliano RP, Ruff CT, Braunwald E, Murphy SA, Wiviott SD, Halperin JL, Waldo AL, Ezekowitz MD, Weitz JI, Špinar J (2013). Edoxaban versus warfarin in patients with atrial fibrillation. N Engl J Med.

[6] Buller HR, Decousus H, Grosso MA, Mercuri M, Middeldorp S, Prins MH, Raskob GE, Schellong SM, Schwocho L, Segers A (2013). Edoxaban versus warfarin for the treatment of symptomatic venous thromboembolism. N Engl J Med.

[7] Lixiana (edoxaban): Summary of Product Characteristics [http://www.ema.europa.eu/docs/en_GB/document_library/EPAR_-_Product_Information/human/002629/WC500189045.pdf]. Accessed 2017.

[8] Konstantinides SV, Torbicki A, Agnelli G, Danchin N, Fitzmaurice D, Galie N, Gibbs JS, Huisman MV, Humbert M, Kucher N, et al: 2014 ESC guidelines on the diagnosis and management of acute pulmonary embolism. Eur Heart J 2014, 35:3033–3069, 3069a-3069k. 10.1093/eurheartj/ehu283 25173341

[9] Summary of the risk management plan (RMP) for Lixiana (edoxaban) [http://www.ema.europa.eu/docs/en_GB/document_library/EPAR_-_Risk-management-plan_summary/human/002629/WC500186050.pdf]. Accessed 2017.

[10] Edoxaban Treatment in Routine Clinical Practice for Patients With Non Valvular Atrial Fibrillation (ETNA-AF-EU: NCT02944019) [https://clinicaltrials.gov/ct2/show/NCT02944019]. Accessed 2017.

[11] Schulman S, Kearon C, the SOCOAOTS, Standardization Committee Of The International Society On T, Haemostasis (2005). Definition of major bleeding in clinical investigations of antihemostatic medicinal products in non-surgical patients. J Thromb Haemost.

[12] Investigators TH-V (2013). Edoxaban versus warfarin for the treatment of symptomatic venous thromboembolism. N Engl J Med.

[13] Cohen AT, Gitt AK, Bauersachs R, Fronk EM, Laeis P, Mismetti P, Monreal M, Willich SN, Bramlage P, Agnelli G (2017). The management of acute venous thromboembolism in clinical practice. Results from the European PREFER in VTE registry. Thromb Haemost.

[14] Agnelli G, Gitt AK, Bauersachs R, Fronk EM, Laeis P, Mismetti P, Monreal M, Willich SN, Wolf WP, Cohen AT (2015). The management of acute venous thromboembolism in clinical practice - study rationale and protocol of the European PREFER in VTE registry. Thromb J.

[15] Raskob G, Buller H, Prins M, Segers A, Shi M, Schwocho L, van Kranen R, Mercuri M (2013). Edoxaban for the long-term treatment of venous thromboembolism: rationale and design of the Hokusai-venous thromboembolism study--methodological implications for clinical trials. J Thromb Haemost.

[16] Guideline on good pharmacovigilance practices (GVP) Module VI – Management and reporting of adverse reactions to medicinal products (Rev 1) [http://www.ema.europa.eu/docs/en_GB/document_library/Scientific_guideline/2014/09/WC500172402.pdf]. Accessed 2017.

[17] Public Policy Committee ISoP (2016). Guidelines for good pharmacoepidemiology practice (GPP). Pharmacoepidemiol Drug Saf.

[18] Verso M, Agnelli G, Ageno W, Imberti D, Moia M, Palareti G, Pistelli R, Cantone V (2012). Long-term death and recurrence in patients with acute venous thromboembolism: the MASTER registry. Thromb Res.

[19] Spirk D, Ugi J, Korte W, Husmann M, Hayoz D, Baldi T, Frauchiger B, Banyai M, Aujesky D, Baumgartner I, Kucher N (2011). Long-term anticoagulation treatment for acute venous thromboembolism in patients with and without cancer. The SWIss venous ThromboEmbolism registry (SWIVTER) II. Thromb Haemost.

[20] Weitz JI, Haas S, Ageno W, Angchaisuksiri P, Bounameaux H, Nielsen JD, Goldhaber SZ, Goto S, Kayani G, Mantovani L (2016). Global anticoagulant registry in the field - venous thromboembolism (GARFIELD-VTE). Rationale and design. Thromb Haemost.

[21] Monreal M, Suarez C, Fajardo JA, Barba R, Uresandi F, Valle R, Rondon P (2003). Management of patients with acute venous thromboembolism: findings from the RIETE registry. Pathophysiol Haemost Thromb.

[22] Ageno W, Mantovani LG, Haas S, Kreutz R, Monje D, Schneider J, van Eickels M, Gebel M, Zell E, Turpie AG (2016). Safety and effectiveness of oral rivaroxaban versus standard anticoagulation for the treatment of symptomatic deep-vein thrombosis (XALIA): an international, prospective, non-interventional study. Lancet Haematol.

[23] Ageno W, Casella IB, Han CK, Raskob GE, Schellong S, Schulman S, Singer DE, Kimura K, Tang W, Desch M, Goldhaber SZ, RE-COVERY DVT (2017). PE: rationale and design of a prospective observational study of acute venous thromboembolism with a focus on dabigatran etexilate. Thromb Haemost.

[24] Ageno W, Mantovani LG, Haas S, Kreutz R, Haupt V, Schneider J, Turpie AG (2014). XALIA: rationale and design of a non-interventional study of rivaroxaban compared with standard therapy for initial and long-term anticoagulation in deep vein thrombosis. Thromb J.

[25] About GARFIELD-VTE [http://vte.garfieldregistry.org/about/about-garfield-vte]. Accessed 2017.

[26] RE-COVERY DVT/PE: Global Study on Treatment Secondary Prevention of Acute Venous Thromboembolism. https://clinicaltrials.gov/ct2/show/NCT02596230.

