# Correction to: Design and rationale of the non-interventional, edoxaban treatment in routiNe clinical prActice in patients with venous ThromboEmbolism in Europe (ETNA-VTE-Europe) study

**DOI:** 10.1186/s12959-018-0173-5

**Published:** 2018-05-31

**Authors:** Alexander T. Cohen, Cihan Ay, Philippe Hainaut, Hervé Décousus, Ulrich Hoffmann, Sean Gaine, Michiel Coppens, Pedro Marques da Silva, David Jimenez, Beatrice Amann-Vesti, Bernd Brüggenjürgen, Pierre Levy, Julio Lopez Bastida, Eric Vicaut, Petra Laeis, Eva-Maria Fronk, Wolfgang Zierhut, Thomas Malzer, Peter Bramlage, Giancarlo Agnelli, on behalf of the ETNA-VTE-Europe investigators

**Affiliations:** 1 Guy’s and St Thomas’ NHS Foundation Trust, King’s College London, London, UK; 2 Clinical Division of Haematology and Haemostaseology, Department of Medicine I, Medical University of Vienna, Vienna, Austria; 3 Department of General Internal Medicine, Cliniques Universitaires Saint Luc, UCL, Bruxelles, Belgium; 4 Centre Hospitalier Universitaire de Saint-Etienne, Saint-Priest En Jarez, France; 5 Division of Angiology, Medical Clinic IV, University Hospital, Ludwig-Maximilians-University, Munich, Germany; 6 National Pulmonary Hypertension Unit, Mater Misericordiae University Hospital, Dublin, Ireland; 7 Department of Vascular Medicine, Academic Medical Center, Amsterdam, The Netherlands; 8 Department of Internal Medicine, Arterial Investigation Unit, Hospital de Santa Marta, Lisbon, Portugal; 9 Respiratory Department, Ramón y Cajal Hospital, Madrid, Spain; 10 Division of Angiology, University Hospital Zurich, Zurich, Switzerland; 11 Institute for Health Economics, Steinbeis-University, Berlin, Germany; 12 LEGOS, Université Paris – Dauphine, Paris, France; 13 University of Castilla-La Mancha, Talavera de la Reina, Toledo Spain; 14 Department of Medicine, Université Paris Descartes, Paris, France; 15 Daiichi Sankyo Europe GmbH, Munich, Germany; 16 Institute for Pharmacology and Preventive Medicine, Berlin, Germany; 17 Internal and Cardiovascular Medicine-Stroke Unit, University of Perugia, Perugia, Italy

## Correction to: Thrombosis Journal (2018) 16:9 DOI 10.1186/s12959-018-0163-7

Following publication of the original article [1], the authors reported that one of the authors’ name was given incorrectly. In this Correction the incorrect and correct author name are shown. The original publication of this article has been corrected.

Originally the author name has been published as:

- David Jimenez Castro

The correct author name is:

- David Jimenez
